# Inner speech deficits in people with aphasia

**DOI:** 10.3389/fpsyg.2015.00528

**Published:** 2015-05-05

**Authors:** Peter Langland-Hassan, Frank R. Faries, Michael J. Richardson, Aimee Dietz

**Affiliations:** ^1^Department of Philosophy, University of CincinnatiCincinnati, OH, USA; ^2^Department of Psychology, University of CincinnatiCincinnati, OH, USA; ^3^Department of Communication Sciences and Disorders, University of CincinnatiCincinnati, OH, USA

**Keywords:** inner speech, aphasia, subvocalization, rhyming, attention, executive function, stroke

## Abstract

Despite the ubiquity of inner speech in our mental lives, methods for objectively assessing inner speech capacities remain underdeveloped. The most common means of assessing inner speech is to present participants with tasks requiring them to silently judge whether two words rhyme. We developed a version of this task to assess the inner speech of a population of patients with aphasia and corresponding language production deficits. Patients’ performance on the silent rhyming task was severely impaired relative to controls. Patients’ performance on this task did not, however, correlate with their performance on a variety of other standard tests of overt language and rhyming abilities. In particular, patients who were generally unimpaired in their abilities to overtly name objects during confrontation naming tasks, and who could reliably judge when two words spoken to them rhymed, were still severely impaired (relative to controls) at completing the silent rhyme task. A variety of explanations for these results are considered, as a means to critically reflecting on the relations among inner speech, outer speech, and silent rhyme judgments more generally.

## Introduction

Inner speech is the little voice in the head, sometimes known as thinking in words. It is the capacity to say things to oneself, silently. When people are asked at random and unexpected intervals to report on the nature of their current conscious experience, they report being engaged in inner speech 20% of the time, on average (Heavey and Hurlburt, 2008).

There are many current proposals concerning the role of inner speech in cognition. One account holds that inner speech is an important element of working memory, with inner speech utterances consisting in recitations within a “phonological loop,” which serves to keep limited amounts of information readily at hand to multiple processing units (Baddeley, 2007). More recently, researchers have found evidence that inner speech underlies certain executive functions, such as the ability to switch between cognitive tasks (Emerson and Miyake, 2003; Vygotsky et al., 2012/1962) and to engage in flexible problem solving of the kind required by the Wisconsin Card Sorting Task (Baldo et al., 2005). A role for inner speech has also been proposed in reading. When people are given information about a speaker’s accent and speaking rate, this influences the rate at which they read text that putatively records that person’s speech (Alexander and Nygaard, 2008; Kurby et al., 2009).

Other views locate inner speech even more centrally within the mind, proposing that a certain kind of thinking is essentially dependent on inner speech. This viewpoint has a long history in psychology, represented by Watson (1930), Paivio (1971, 1986) and Vygotsky et al. (2012/1962). More recently, Carruthers (2002) has argued that inner speech allows us to collect and integrate information from a variety of cognitive modules; and Gauker (2011) has proposed that all thought that deserves to be called *conceptual thought* occurs in inner speech. A number of theorists have also proposed a role for inner speech in metacognition (thinking about one’s own thinking) and self-awareness (Jackendoff, 1996; Clark, 1998; Bermudez, 2003; Morin, 2009; Carruthers, 2011).

One of the most effective ways to assess proposals concerning the role of inner speech in cognition is to compare the abilities of people with impaired or absent inner speech to those whose inner speech is intact. But how can we know whether, and to what degree, someone is able to generate inner speech? How can we assess, in a scientifically rigorous way, whether someone has the ability to say things to him or herself silently? Historically, the primary means for such an evaluation has been to present participants with pairs of words and to ask them to judge, silently, whether the words rhyme (e.g., “chair” and “care”), or whether they are homophones (e.g., “stare” and “stair”; Levine et al., 1982; Feinberg et al., 1986; Geva et al., 2011a,b). Such studies use a high proportion of target words-pairs that rhyme but which do not have similar endings (e.g., “box” and “socks”), to prevent participants from answering based on the words’ visually apparent orthography. Occasionally non-words are used as stimuli (e.g., “pole” and “voal”) to prevent participants from answering based on knowledge of orthography (see, e.g., Geva et al., 2011a,b). Alternatively, pairs of pictures may be used as stimuli, with participants being asked to indicate whether the words for the pictured objects rhyme (Sasisekaran et al., 2006). This approach reduces possible interference by difficulties a participant may have with reading or with language reception generally.

Intuitively, we make silent rhyme or homophone judgments by uttering the relevant words in inner speech. However, inner speech cannot simply be defined operationally as the ability to silently make correct judgments about the sounds of words. After all, a person who accurately *guesses* whether a pair of words rhyme would not in virtue of that success count as having used inner speech. And, moreover, it is possible that a person may be able to silently utter words in inner speech yet nevertheless be unable to accurately judge whether they rhyme. However, there are at present no better-established or more reliable means for assessing inner speech abilities.

The present study seeks to better understand the relationship between inner speech and the ability to silently judge rhymes, by assessing the relationship between outer speech and silent rhyme judgments in a population with post-stroke aphasia. People with aphasia (PWA) have impaired language capacities due to one or more neural lesions, typically acquired as a result of stroke. Depending on the location of the lesion, a patient’s deficits may center more on the production of speech (as in Broca’s, conduction, and anomic aphasia), or on the comprehension of speech (as in Wernicke’s aphasia). However, almost all PWA have at least some difficulties with respect to both speech production and comprehension.

People with aphasia have been shown in many cases to have impaired inner speech (Levine et al., 1982; Feinberg et al., 1986; Geva et al., 2011a,b). Yet there is relatively little data available concerning the precise relationship between outer speech and inner speech abilities in PWA. Nor, for that matter, is there much data available on the relation between inner and outer speech abilities in the general population. Geva et al. (2011a) offer the most thorough examination of the relation between inner and outer speech in PWA. Their work confirms that PWA are in general significantly impaired at silent rhyme and homophone tasks, compared to controls. They note, however, a high degree of variability in inner speech abilities, with some patients performing at normal levels, while others are clearly impaired.

Geva et al. (2011a) also sought to assess the relationship between inner and outer speech abilities in PWA. Their main task required patients to read pairs of words or non-words both silently (in one condition) and out loud (in another). In one condition, they were asked to judge whether the two words rhymed; in a second they were asked to judge whether the two words were homophones; and, in a third, they were asked to judge whether the two non-words were homophones. In each case the judgments of PWA during the silent condition were impaired relative to controls. And while significant correlations were found between the inner and outer speech abilities of PWA, their data also revealed a number of interesting dissociations in individual patients. Some patients showed relatively strong performance in the silent versions of the task, yet were impaired on the overt versions; this suggests intact inner speech with impaired overt speech. And, perhaps more surprisingly, dissociations were also found in the opposite direction, with some participants showing impairments in the silent rhyme judgments, yet normal performance in the overt rhyme judgment tasks. This in turn suggests impaired inner speech in the presence of relatively normal overt speech. While reports of this dissociation are rare, there are precedents (Levine et al., 1982; Papafragou et al., 2008). Moreover, the rarity of such reports may be due in part to the fact that inner speech capacities are seldom assessed in people with relatively intact overt speech.

In light of these somewhat surprising dissociations, Geva et al. (2011a) urge that more data should be collected concerning the inner speech abilities of PWA. The present study, conducted as part of a larger investigation of the relation between inner speech and metacognition (under review), is reported with that in mind. From a therapeutic standpoint, if deficits in overt speech are not always accompanied by deficits in inner speech, this could open the door to therapeutic interventions that make use of a patient’s preserved inner speech. Arguably, assessments of inner speech should then become a standard component of aphasia screening tests. And, from a more theoretical perspective, if there are significant dissociations between outer speech capacities and capacities to silently judge rhymes, this may call into question the links many presume between inner speech and outer speech. Specifically, it may challenge a common view of inner speech as overt speech minus a motor component (Oppenheim and Dell, 2010; Carruthers, 2011; Pickering and Garrod, 2013). At the same time, dissociations between outer speech and the ability to silently judge rhymes may lead us to consider more carefully the possibility that silent rhyme judgments require something more than intact inner speech.

In the present study, we first sought to confirm whether PWA would show deficits compared to controls in a pictorial silent rhyme judgment task that [unlike Geva et al. (2011a)] did not require them to read words. A second interest was in whether the performance of PWA on the silent rhyme task would be correlated with their ability to judge whether words overtly spoken to them rhyme. In this way, we sought to understand the relation between judging rhymes, in general, and judging rhymes silently. A third interest was to investigate what correlations may exist between the silent rhyming abilities of PWA and their abilities on various cognitive and linguistic tests of the Western Aphasia Battery-Revised (WAB-R; Kertesz, 2006) and Cognitive Linguistic Quick Task (CLQT; Helm, 2003), including tests of confrontation naming abilities, generative naming abilities, executive function, and attention. In this way we sought to better understand the relation of the capacity for making silent rhyme judgments to an array of other seemingly closely related abilities.

## Materials and Methods

### Participants

#### Patients with Aphasia

A total of 11 individuals (4M/7F, 10 right-handed, one left-handed, mean age 60.3 ± 8.0, age range 44–76, mean years of education 15.3 ± 2.1) with chronic post-stroke aphasia (mean months post-stroke=124 ± 77.9) were recruited from a registry of patients held at the University of Cincinnati College of Allied Health Sciences, Division of Communication Sciences and Disorders^1^. All were native English speakers. A diagnosis of aphasia was confirmed based on their performance on the Western Aphasia Battery – Revised (Kertesz, 2006). All PWA received a diagnosis of Conduction, Broca’s, or Anomic aphasia (see **Table 1**), and all exhibited significant difficulties with language production. Their language comprehension capacities, however, were relatively intact, allowing them to given informed consent and to understand task instructions.

**Table 1:** Demographic information, participants with aphasia.

	Sex	Age	Years of education	Months post-onset	Aphasia type
201	Female	59	14	72	Conduction
202^1^	Male	44	16	112	Broca’s
203	Male	58	18	76	Anomic
204^2^	Female	68	16	175	Broca’s
206	Female	76	14	315	Broca’s
207	Female	56	14	92	Broca’s
208	Female	54	12	134	Anomic
209	Male	60	16	101	Broca’s
210	Female	67	18	100	Broca’s
211	Female	62	16	172	Conduction
213	Male	59	14	15	Broca’s

*^1^Apraxia of Speech present based on clinical judgment*.

*^2^Left handed pre-stroke*.

#### Control Participants

A total of 12 healthy volunteers (4M/8F, mean age 58.7 ± 8.5, age range 47–78, mean years of education 14.8 ± 1.7) were chosen for a control group, roughly matched in age [*t*(21) = 0.47, *p* > 0.64], gender, and education [*t*(21) = 0.55, *p* > 0.58] to the PWA. All were native speakers of English, with no history of stroke or other neurological or psychiatric disorders.

### Materials

All PWA were administered basic vision and hearing screening exams, the WAB-R (Kertesz, 2006), and the CLQT (Helm, 2003). An overt rhyme judgment task (described below) was also administered to PWA.

For the silent rhyming task, 88 digital photographs and drawings were used to create 44 trials involving two pictures each. These trials were presented on an Asus 8A-Series computer with a 21-inch touch-sensitive screen. The program was written in C++ and recorded responses and response times automatically (10 ms resolution).

### Procedure

In the first session, all PWA completed the WAB-R and CLQT tests, as well as basic hearing and vision screening tests. The WAB-R was used to confirm aphasia severity and type, and to assess overt word production abilities. Of particular interest was participant performance on confrontation naming tasks (where objects are shown to the participant and the participant must name them), and generative naming tasks (where a category, such as “animals,” is given to participants and they must name as many members of that category as possible). The CLQT also included confrontation and generative naming tasks, together with a variety of non-linguistic cognitive tests that were used to rule out the possibility that task performance was due to cognitive limitations unrelated to the patients’ language deficits.

At the beginning of the second session, the overt rhyme judgment task was administered to PWA. During this task, 10 pairs of one-syllable words were spoken aloud by the experimenter, with patients indicating (Yes or No) whether the words rhymed. The mean word frequency for the words used (using log frequency sore) was 1.75 (SD = 0.51; Medler and Binder, 2005). This task was administered in order to investigate the relation between judging rhymes that are heard and judging rhymes silently, through inner speech.

After completing the overt rhyming task, PWA were administered the silent rhyming task. Controls were tested for only a single session, and were not administered the overt rhyming task, the WAB-R, or the CLQT. Controls were not administered these tests mainly because they were not relevant to the larger study from which this report is retrospectively made.

**FIGURE 1:**
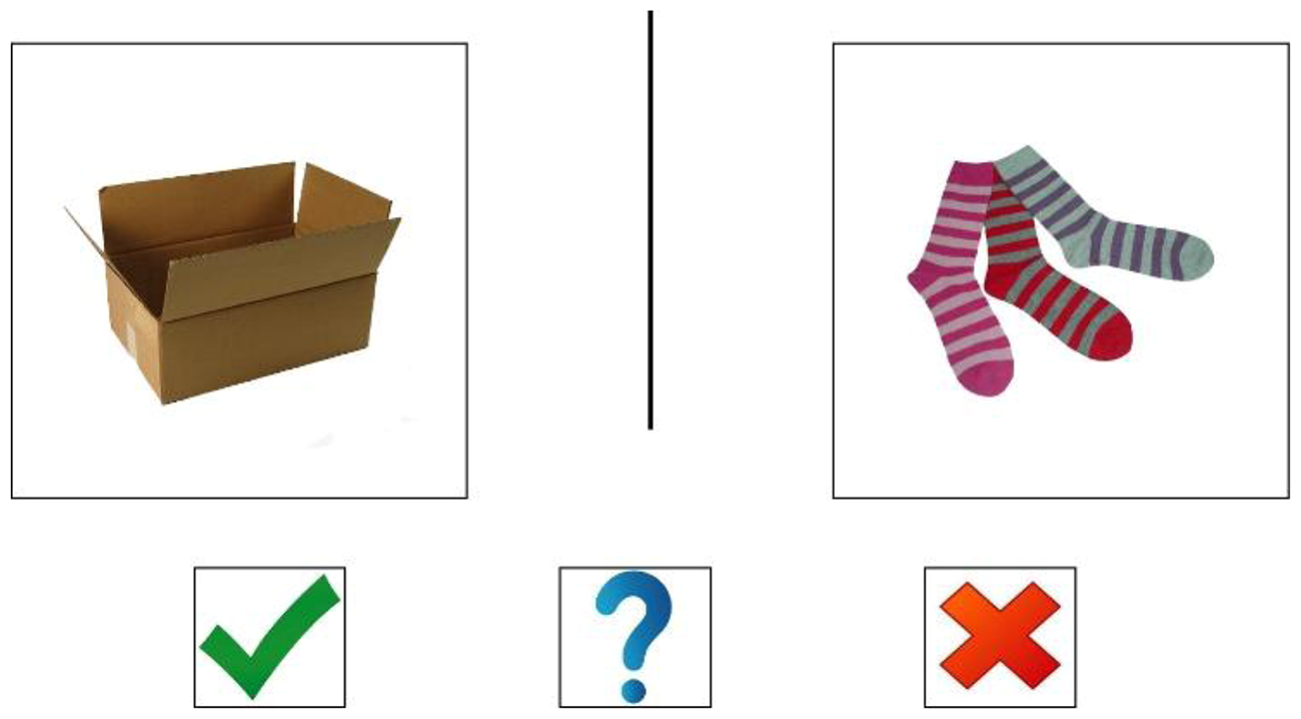
**Example of a silent rhyming task trial**.

The silent rhyming task was administered as follows. On a touchscreen computer, participants were shown 44 sets of two pictures each, one set at a time, and were asked to indicate, without speaking aloud, whether the words for the pictured items rhyme (**Figure 1**). The first four trials were training trials, during which a team of experimenters demonstrated the task by first completing it out loud, and then silently. The experimenters read from a script to ensure that the task was explained in the same way for all participants. During training trials, it was emphasized that participants must answer the questions without making any vocal sounds. In addition to answering yes (by touching a green check) or no (by touching a red X), participants could indicate that they did not know by touching a blue question mark. Touching the blue question mark was counted as an incorrect answer, for purposes of scoring. Among the 40 test trials, 20 presented stimuli that rhymed, while 20 did not. Of the 20 rhyming trials, 10 involved pictures of items whose linguistic labels rhymed but did not share similar orthographic endings (e.g., “box” and “socks”). This was to decrease the likelihood that participants could answer by forming visual images of written words as opposed to using auditory-phonological cues. The mean word frequency rating (using the log frequency score) for the words tested was 1.38 (SD = 0.87; Medler and Binder, 2005)^2^.

## Results

### Silent Rhyming Impairment in PWA

**FIGURE 2:**
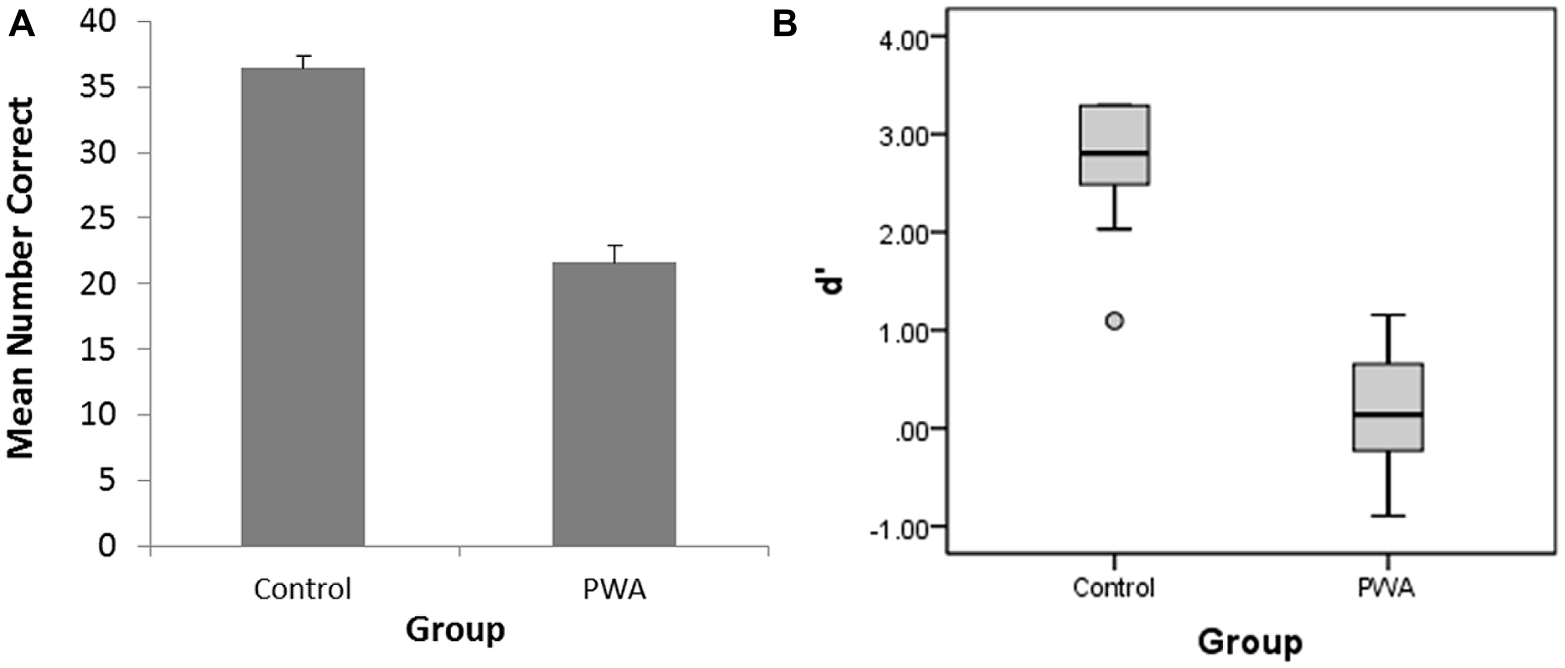
**(A)** Number of correct responses (hits) on the silent rhyming task for people with aphasia (PWA) and controls; error bars correspond to standard deviations of the mean. **(B)** Box plot of the *d*-prime scores for PWA and controls.

An independent-samples *t*-test was conducted to compare silent rhyming task performance (i.e., hits) in control and PWA conditions. This analysis revealed a significant difference between controls (mean = 36.4, SD = 3.29) and PWA (mean = 21.64, SD = 4.30); *t*(21) = 9.32, *p* < 0.001. There was also a significant difference between controls and PWA with regard to *d*-prime, *t*(21) = 9.18, *p* < 0.001, (see **Figure 2**), with PWA essentially guessing when providing their answers on the silent-rhyme task. (*d*-prime is a sensitivity index from signal detection theory which, for the present study, captured the ability of participants to discriminate or detect whether two words rhymed.) Notably, the average number of false alarms (touching the check mark or opting out when there was not a rhyme) for PWA was 10.18 (SD = 4.56), compared to 0.58 (SD = 1.165) for controls [*t*(21) = 7.06, *p <*0.001]. There was also a similarly large difference between the average number of misses (touching the red X or opting out when there was a rhyme) for PWA (mean = 8.18, SD = 3.92) compared to controls [mean = 3.0, SD = 2.45; *t*(21) = 3.84, *p* < 0.01]. **Table 2** shows the respective means for PWA and controls on the silent rhyming task with respect to hits, correct rejections, false alarms, misses, opt-outs, and *d*-prime. Finally, the mean number of PWA who correctly identified a rhyming pair as rhyming was not significantly higher when the orthography of the word-endings matched (mean = 7.6; ±1.43) than when the word-endings did not orthographically match [mean = 7.2; ±1.23; *t*(9) = -0.684, *p* > 0.51].

**Table 2:** Mean scores by population on silent rhyming task.

	People with aphasia (PWA)	Controls
Hits^1^	11.8	17.0
Correct rejections^2^	9.8	19.4
Misses^3^	8.2	3.0
False alarms^4^	10.2	0.6
Opt-out^5^	1.7	0.6
*d*-prime	0.2	2.7

*^1^Hits = touching green check for rhyming pairs*.

*^2^Correct rejections = touching red X for non-rhyming pairs*.

*^3^Misses = touching red X or opting-out when pair in fact rhymed*.

*^4^False alarms = touching green check or opting out when pair did not rhyme*.

*^5^Opt-out = touching blue question mark*.

### Lack of Correlation Between Silent-Rhyming and Overt Rhyming

On the overt rhyme task, the performance of PWA was considerably better than their performance on the silent rhyme task, with a mean score of 8.67 out of 10 (SD = 1.2). Spearman’s Rank order correlations were assessed with respect to the silent rhyming scores of PWA and their scores on the overt rhyme task (see **Table 3**). For PWA, the correlation between silent rhyme performance and overt rhyme performance fell short of significance (*r* = 0.443, *p* > 0.17). Possible reasons for this null effect are discussed below (Controls did not complete the overt rhyme task, for reasons discussed above.).

**Table 3:** Raw scores of participants with aphasia on rhyming, WAB-R, and CLQT tests.

ID#	23pc Silent rhyming	Overt rhyming	Western Aphasia Battery-Revised (WAB-R) aphasia quotient	WAB-R object naming	WAB-R word fluency	Cognitive Linguistic Quick Task (CLQT) confrontation naming	CLQT generative naming	CLQT attention^5^	CLQT executive functions^6^
Max^1^	40	10	100	60	20	10	Unlimited	215	40
Min^2^	NA^3^	NA	93.8	NA	NA	10	5	180	24
201	17	9	81.8	51	9	9	1	197	37
202	28	9	71.2	55	11	10	3	191	30
203	22	10	68.9	30	7	7	2	189	27
204	26	8	43.9	26	1	6	1	180	20
206^4^	20	9	66.1	47	5	10	2	137	10
207	17	6	50.6	36	7	8	2	199	29
208	27	10	73.4	57	3	10	1	153	22
209	15	7	70.9	50	10	10	3	182	28
210	24	8	30.2	0	0	2	0	170	20
211	21	9	81.1	55	13	9	4	161	23
213	21	9	59.2	33	4	9	1	185	23

*^1^Maximum possible scores are indicated for each subtest*.

*^2^Minimum score for normal performance. Any lower score is considered impaired*.

*^3^The mean score for controls on this test was 36.4*.

*^4^Minimum score of normal performance on CLQT is lower for this participant, given age (see note 5)*.

*^5^Ranges for participants up to 69 years old: within normal limits = 215–180, Mild = 179–125, Moderate = 124–50, Severe = 49–0; for participants over 70 years and older: within normal limits: 215–160, Mild: 159–100, Moderate: 99–40, Severe: 39–0*.

*^6^Ranges for participants up to 69 years old: WNL: 40–24, Mild: 23–20, Moderate: 19–15, Severe: 14–0; for participants 70 years and older: WNL 40–19, Mild: 18–14, Moderate: 13–8, Severe 7–0*.

### Lack of Correlations Between Silent Rhyming and Sub-Tests of CLQT and WAB-R

The CLQT and WAB-R tests revealed the following impairments in PWA. On the CLQT, four out of 11 fell below the cut-off score for normal performance (as established and validated by the designers of the CLQT) on sub-tests for Attention (mean = 176.7, SD = 19.5), while six of 11 fell below the cut-off score for normal performance on sub-tests for Executive Functions (mean = 24.5, SD = 6.8). Seven out of 11 fell below normal limits on the CLQT confrontation naming task (mean = 8.2, SD = 2.4), while 11 out of 11 fell below normal limits on the CLQT generative naming task (mean = 1.8, SD = 1.2). The mean score of PWA with respect to object naming on the WAB-R was 40, out of a possible 60 (SD = 17.2), while the mean score of PWA on the WAB-R word fluency test was 6.4, out of a possible 20 (SD = 4.2) (Controls did not complete the WAB-R or CLQT, for reasons discussed above.).

No significant correlations were found between the silent rhyming performance of PWA and the performance of PWA on these sub-tests of the WAB-R and CLQT (all *p* > 0.49; see **Table 4**). For instance, the correlation between PWA silent rhyming performance and the WAB-R Object Naming task was only *r* = -0.030; and the correlation between PWA silent rhyming scores and their CLQT confrontation naming scores was *r* = -0.104. Nor were there significant correlations found between PWA silent rhyming scores and Executive Function (*r* = -0.294) or Attention (*r* = -0.242) scores on the CLQT.

**Table 4:** Between measure correlations (Spearman’s rank order correlation).

	Silent rhyming	d′	Overt rhyming	WAB aphasia quotient	WAB object naming	WAB word fluency	CLQT confrontation naming	CLQT generative naming	CLQT attention	CLQT executive function
SR	-	0.991 (0.000)	0.443 (0.173)	-0.100 (0.769)	-0.030 (0.931)	-0.336 (0.312)	-0.104 (0.761)	-0.232 (0.473)	-0.242 (0.473)	-0.294 (0.381)
d′	0.991 (0.000)	-	0.475 (0.139)	-0.118 (0.729)	-0.018 (0.957)	-0.432 (0.185)	-0.104 (0.762)	-0.323 (0.332)	0.287 (0.392)	-0.348 (0.295)
OLR	0.443 (0.173)	0.475 (0.139)	-	0.527 (0.096)	0.379 (0.250)	0.046 (0.894)	0.275 (0.413)	-0.025 (0.942)	-0.254 (0.451)	-0.067 (0.844)
WAB aphasia quotient	-0.100 (0.769)	-0.118 (0.729)	0.527 (0.096)	-	0.866 (0.001)	0.697 (0.017)	0.606 (0.048)	0.438 (0.178)	0.036 (0.915)	0.507 (0.112)
WAB object naming	-0.030 (0.931)	-0.018 (0.957)	0.379 (0.250)	0.866 (0.001)	-	0.607 (0.048)	0.803 (0.003)	0.488 (0.127)	-0.100 (0.769)	0.373 (0.259)
WAB word fluency	-0.336 (0.312)	-0.432 (0.185)	0.046 (0.894)	0.697 (0.017)	0.607 (0.048)	-	0.461 (0.153)	0.868 (0.001)	0.342 (0.304)	0.687 (0.020)
CLQT confrontation naming	-0.104 (0.761)	-0.104 (0.762)	0.275 (0.413)	0.606 (0.048)	0.803 (0.003)	0.461 (0.153)	-	0.479 (0.136)	-0.216 (0.523)	0.179 (0.598)
CLQT generative naming	-0.232 (0.493)	-0.323 (0.332)	-0.025 (0.942)	0.438 (0.178)	0.488 (0.127)	0.868 (0.001)	0.479 (0.136)	-	0.071 (0.837)	0.355 (0.284)
CLQT attention	-0.242 (0.473)	0.287 (0.392)	-0.254 (0.451)	0.036 (0.915)	-0.100 (0.769)	0.342 (0.304)	-0.216 (0.523)	0.071 (0.837)	-	0.854 (0.001)
CLQT executive function	-0.294 (0.381)	-0.348 (0.295)	-0.067 (0.844)	0.507 (0.112)	0.373 (0.259)	0.687 (0.020)	0.179 (0.598)	0.355 (0.284)	0.854 (0.001)	-

Significant correlations were, however, found between certain subtests of the WAB-R and CLQT (see **Table 4**). For instance, the scores for PWA on the WAB-R object naming were highly correlated (*r* = 0.803) with their scores on the CLQT confrontation naming task. And the scores for PWA on the WAB-R word fluency task were highly correlated (*r* = 0.868) with their scores on the CLQT generative naming task.

## Discussion

In this study we sought to assess the degree to which the inner speech of people with known outer speech deficits (due to aphasia) is impaired, relative to controls. We also sought to assess the degree of correlation between their inner speech impairments and their overt speech and rhyming abilities. And, finally, we wanted to investigate what correlations there might be between their inner speech abilities and their aptitude on measures of executive function and attention. In this way, we hoped to gain a clearer understanding of the relation between inner and outer speech, and between inner speech and executive function and attention. Currently, the degree to which inner speech is a distinct mental capacity, dissociable from these others, is not well understood.

Our main finding was that PWA (with, specifically, Broca’s, conduction, and anomic aphasia) have great difficulty completing silent rhyming tasks, compared to controls. Insofar as performance on the silent rhyming task is a reliable indicator of inner speech ability, their inner speech was severely impaired. More surprisingly, however, we did not note any significant correlations between their silent rhyming abilities and their abilities on various overt rhyming and overt speech tasks. We make note of these and other null effects—and discuss them further below—not because any strong inferences can be made from the lack of such correlations, but because they are of interest in considering possibilities for future research. In particular, they raise interesting questions concerning the degree to which inner speech may be a distinct capacity, dissociable both from overt speech and from cognitive capacities such as executive function and attention.

The deficits of PWA on the silent rhyming task compared to controls were even more pronounced than those found by Geva et al. (2011a), whose patients with aphasia answered approximately 80% of silent rhyme and homophone judgment prompts correctly. In the present study, PWA answered only 54% of silent rhyme questions correctly, and had a high proportion of false alarms [mean = 10.18 (±4.56)]. Such results would be expected if participants were simply unable to perform the task and were guessing on each trial.

One might hypothesize that the greater difficulty shown by patients in the present study resulted from their having to find the proper word corresponding to each image before generating the words in inner speech. In the case of Geva et al. (2011a) the words were given to patients in written form, obviating the need to find the proper word for the objects. However, the data recorded on overt naming abilities in this group of PWA does not support this interpretation. For if the relative difficulty of the silent rhyming task was to be explained by a general inability of patients to generate words for the pictured items, we would expect patients to show difficulties *both* with the silent rhyming task and with ordinary confrontation naming tasks. For both kinds of task confront the participant with objects (or pictures of objects) and require that they name them, either in inner speech, or overtly. Yet, as evidenced by the scores of PWA on the WAB object naming task and the CLQT confrontation naming task (both of which are confrontation naming tasks) many of the patients showed relatively intact confrontation naming abilities, with several performing within normal limits (see **Table 3**). So the severity of the silent rhyme deficits observed, compared to Geva et al. (2011a), might not be due to general difficulties generating words for presented objects. Indeed, while the PWA showed a broad continuum of abilities on the confrontation naming tasks, there was not a significant correlation between those abilities and their scores on the silent rhyming task. Yet one would expect there to be such a correlation if an inability to succeed at confrontation naming (both inner and overt) explained their difficulties. Moreover, the mean word frequency ratings for the words featured in the CLQT object naming task (mean = 1.36; ±0.57) and WAB confrontation naming task (mean = 1.26; ± 0.70) are lower than for those featured in the silent rhyming task (mean = 1.38; ± 0.87). The better performance of PWA on the overt naming tasks than silent rhyming task is therefore not a result of the silent rhyme task using less familiar words^3^.

If a general inability to name objects does not explain the poor performance of PWA on the silent rhyming task, what does? One possibility is that, while the PWA were able to generate the words for the pictured items in inner speech, they were unable to reliably judge whether the words rhyme, due to a specific deficit in discriminating rhyming words from non-rhyming words. However, the data do not support this interpretation. The abilities of PWA to judge rhymes when word pairs were spoken to them by the experimenter was relatively intact, with 87% of their answers being correct, as compared to only 54% for the silent rhyming task. It should be noted, however, that the mean word frequency of the words used in the overt rhyming task (1.75, ±0.51) was significantly higher [*t*(96) = 2.55, *p* = 0.012] than that of the words used in the silent rhyming task (1.38, ±0.87). It is possible that the increased familiarity of the words spoken aloud to participants played some role in facilitating their ability to judge whether the words rhymed. Nevertheless, it is less obvious in this case, as compared to the confrontation naming tasks, why word frequency would influence performance. Participants did not, after all, have to generate the relevant words for the overt rhyming task; they only had to listen to the words and judge whether they rhymed. Nor was there a significant correlation observed between overt rhyming scores and silent rhyming scores. Indeed, eight out of the 11 patients had scores of either 9 or 10 (out of 10) on the overt rhyming task (**Table 3**); yet the mean score among those same eight patients on the silent rhyming task was 22.3 (out of 40), or only 56% correct. Thus, it is not clear that the poor performance exhibited by PWA on the silent rhyming task, compared to controls, can be explained by a general impairment in judging aurally presented rhymes. That said, it is worth bearing in mind the *r* = 0.443 correlation found between silent rhyming scores and overt rhyming scores, for the PWA (**Table 4**). While not statistically significant, this degree of correlation in our low-powered sample warrants further investigation of the link between these two abilities. A possibility worth bearing in mind is that even judging aurally perceived rhymes requires some degree of instantaneous “replay” of the words in inner speech. In that case, an inability to judge whether aurally perceived word pairs rhyme could be explained in terms of an inability to generate words in inner speech, and not vice versa.

Nevertheless, in our sample, patients could reliably judge whether words spoken to them rhymed, and were in many cases relatively unimpaired at overtly naming pictured objects, yet were without exception unable to complete the silent rhyme task comparably to controls^4^. There are at least two ways to interpret this finding. First, it could be that the preserved ability of some patients to *overtly* name objects with which they are confronted was not matched by a comparable ability to generate the names for objects *in inner speech*. This would be a surprising finding in light of theories that conceive of inner speech as motor-precursor to outer speech (Oppenheim and Dell, 2008; Pickering and Garrod, 2013). Yet it may be less surprising from a Vygotskian perspective, which views inner speech as an internalized, and developmentally posterior, version of outer speech (Jones and Fernyhough, 2007; Vygotsky et al., 2012/1962). The hypothesis that inner speech deficits were not matched by comparable overt naming deficits meshes with a handful of other studies that have reported intact language in the absence of inner speech (Levine et al., 1982; Papafragou et al., 2008; Geva et al., 2011a), and with a recent study showing distinct neural correlates for inner and outer speech (Geva et al., 2011b).

An alternative hypothesis is that, while the PWA had no deficits in using inner speech to inwardly name the pictured objects (compared to their overt confrontation naming abilities), and no severe impairments judging rhymes when they were heard, nevertheless the task as a whole presented a cognitive load that was too great to overcome, given their impairments. That is, perhaps silently judging rhymes in inner speech requires working memory resources, or executive function abilities, that PWA lack. To be clear, this hypothesis holds that the specific resource lacked is not that which makes it possible to utter the relevant words in inner speech, or to judge rhymes in general. The idea is that it may be something else, such as the resource that makes it possible to hold two words in mind long enough to judge whether they rhyme (i.e., working memory), or that which allows one to assign requisite attention to the task (e.g., executive function). It should be noted, however, that the PWA did not in general show significant deficits in executive functions or attention, on the CLQT. With respect to executive functions, five out of 11 scored within normal limits, with five having only mild impairments, and one having moderate impairments (see **Table 3**). And, with respect to attention, seven of 11 scored within normal limits, with the other four being only mildly impaired. Nor were there any significant correlations between silent rhyming scores and executive function or attention scores on the CLQT. Moreover, it should be noted that some of both the attention and executive functions sub-tests explicitly required language use, which was of course known to be impaired in these participants. Their relatively strong cumulative scores for executive functions and attention therefore suggest that they did not have considerable cognitive impairments *outside of* their specific linguistic deficits. It is therefore unclear what sort of general, non-linguistic cognitive deficit might have accounted for the special difficulties PWA had, compared to controls, on the silent rhyming task. Another possibility is that the CLQT is not sensitive to the reduced ability to allocate attention to language-related tasks because it relies largely on non-verbal cognitive tasks. It is well accepted in aphasiology that aphasia can be explained as a deficit in resource allocation, specifically for language (while other aspects of cognition are intact; Murray, 1999). For now it remains possible that the capacity to generate inner speech is simply a distinct ability from executive function, attention, outward rhyme judging, and overt naming—one with its own neural substrate, and which can be severely impaired without comparable impairments in these other capacities.

Future work could further tease apart these hypotheses by using other implicit measures of inner speech on a similar population. For instance, studying populations whose native languages encode information about bounded motion differently, Papafragou et al. (2008) found that the way a speaker’s native language encodes such information influences their eye movements when surveying an action sequence that they are told to remember. This suggests that inner speech may be influencing visual search in such cases. If the eye movements of PWA did not, under such conditions, match the patterns typical of controls with the same native language, this would be corroborating evidence that they did not in fact have intact inner speech (even if their outer speech was comparably preserved). It bears noting, however, that language-learning may affect thought by influencing the way in which non-linguistic thought structures develop, and not necessarily through the mediation of inner speech.

Whatever the explanation for the low scores of PWA on the silent rhyming task, it is certainly of interest to note that no significant correlations were found between the various language and cognitive assessment scores patients received on the WAB-R and CLQT, and their scores on the silent rhyming task. By contrast, patient scores on the WAB and CLQT tests were often highly correlated with each other, as one would expect (see Results; **Table 4**). One reason for the lack of correlations may simply be due to the relatively small sample size (i.e., low power). An additional explanation for the lack of correlations may be that all, or almost all, of the PWA were simply incapable of completing the silent rhyme task. The highest score of any patient on the task was 28 (out of 40), which was over 2.5 SDs below the mean for controls; and the mean score for PWA was barely above 50% correct. Furthermore, the mean *d*-prime score for PWA on the silent rhyming task was only 0.2, which is little better than what would be expected (0.0) if they were all guessing all of the time. Thus, their ability, as a group, to discriminate rhyming trials from non-rhyming trials was sufficiently low to render unlikely any meaningful correlations with their more widely distributed scores on the WAB-R or CLQT.

Left unanswered, however, is why the patients whose aphasia was mild by comparison to others and who scored at normal or near-normal levels on some language production tasks (e.g., 201, 202, 211) did not have comparably better scores on the silent rhyming task. A further datum worth noting in this regard is that our population of PWA all had significant deficits on the generative naming components of the WAB-R and CLQT (The WAB-R generative naming task is called “Word Fluency,” and the CLQT generative naming task is called “Generative Naming” on **Table 3**.). Only one participant (211) had scores on either test that approached levels typical for controls. Thus we did not observe the same kind of dissociation between generative naming abilities and silent rhyming abilities as we did between confrontation naming and silent rhyming. It is not immediately obvious why severe impairments in silent rhyming would co-occur with severe impairments in generative naming, as opposed to confrontation naming.

One possible explanation is that, on the CLQT and WAB-R confrontation naming tasks, participants were not in the situation of having to choose which of several common names to give for the pictured objects. This is because the CLQT counts any commonly used name that is produced for a pictured item as a correct response, while the WAB-R is designed so as to only feature stimuli with one common name. By comparison, the silent rhyming task has some of the character of a generative naming task, to the extent that participants may have had to audition (and therefore generate) multiple appropriate names for a single object in order to identify whether each trial was a rhyming trial. When faced, for instance, with a picture of a box and a pair of socks, a participant’s success may have required moving past a first word generated in response to the stimulus (e.g., “package”), to find another (e.g., “box”) that rhymed with the word for the companion picture (e.g., “socks”). If this were the case, it would help explain why serious deficits in generative naming went hand-in-hand with troubles on the silent rhyming task^5^. That said, there were no statistically significant correlations between performance on the generative naming tasks and performance on the silent rhyming task. It would be useful, in future work, to investigate whether clearer dissociations or correlations can be found between silent rhyming abilities and generative naming abilities, by ensuring that the stimuli used in silent rhyming tasks do not require or encourage participants to potentially generate multiple words in response to the stimulus. One way to do this would be to use pairs of written words as stimuli, as in Geva et al. (2011a,b).

## Conclusion

Our participants with aphasia showed severe deficits at the kinds of silent rhyming tasks that are typically used to assess inner speech abilities. This suggests that PWA with impaired overt speech typically have impaired inner speech as well. Interestingly, even patients with relatively preserved confrontation naming and overt rhyming abilities performed at chance on the silent rhyming task. This highlights the possibility that inner speech abilities are often more severely impaired in PWA than overt speech capacities. While more research must be done before any strong conclusions can be made, it may simply be that generating and using inner speech is more cognitively and linguistically demanding than generating overt speech and, further, that the neural substrates for each are somewhat distinct (Geva et al., 2011b). This would mesh well with a Vygotskian perspective on which inner speech develops posterior to overt speech, and is by comparison a more sophisticated and cognitively demanding activity.

## Conflict of Interest Statement

The authors declare that the research was conducted in the absence of any commercial or financial relationships that could be construed as a potential conflict of interest.

## Footnotes

1 A 12th person with a history of aphasia was recruited and tested, but excluded from analysis because his Aphasia Quotient (=96) on the WAB-R was not sufficiently low to qualify him for a diagnosis of aphasia.

2 The word frequency ratings for the silent rhyming words were calculated after removing ‘can’ from the group. ‘Can’ was an outlier in having an extremely high frequency rating, due to its common use as an auxiliary verb (The McWord database is only sensitive to orthographic form). The silent rhyming test, however, made use of ‘can’ in its noun sense, showing a picture of a can—hence its exclusion.

3 A reviewer raises the possibility that the better performance of patients on silent rhyming tasks in Geva et al. (2011a) compared to the present study may have been due to the potentially greater word-frequency of the words used by Geva et al. (2011a). Geva et al. (2011a) do not report the word frequency ratings for their stimuli, nor the specific words used, so we cannot assess this possibility.

4 The highest score of any patient on the silent rhyme task was over 2.5 SD below the mean for controls on that task.

5 We thank a reviewer for raising this possibility.
